# Effects of a lifestyle intervention on the biomarkers of oxidative stress in non-communicable diseases: A systematic review

**DOI:** 10.3389/fragi.2023.1085511

**Published:** 2023-03-09

**Authors:** Sarah Husain, Katharina Hillmann, Karin Hengst, Heike Englert

**Affiliations:** ^1^ Faculty of Medicine, University of Muenster (WWU), Münster, Germany; ^2^ Department of Food, Nutrition and Facilities, University of Applied Sciences Muenster, Münster, Germany; ^3^ Department of medicine, University hospital Muenster (UKM), Münster, Germany

**Keywords:** oxidative stress, lifestyle intervention, lifestyle diseases, antioxidants, non-communicable diseases, prevention, ageing, immunity

## Abstract

Oxidative stress plays a critical role in the pathogenesis of chronic diseases. Therefore, improvement of oxidative stress status through lifestyle intervention can play a vital role in preventing and treating chronic diseases. This systematic review aims to provide an overview of articles published in the last decade examining the association between lifestyle intervention and oxidative stress biomarkers in the context of non-communicable diseases. The electronic databases PubMed and Web of Science were searched for relevant studies, following the PRISMA (Preferred Reporting of Systematic Reviews and Meta-Analyses) guidelines. This systematic review focused on the four important oxidative stress biomarkers; glutathione (GSH), superoxide dismutase (SOD), catalase, and malondialdehyde. 671 articles were identified, of which nine met the inclusion criteria. A trend emerged, showing that lifestyle modifications that focus on diet and physical health can improve oxidative stress in the form of an increase in superoxide dismutase and CAT levels and a decrease in Malondialdehyde levels in participants with non-communicable diseases (NCDs), GSH levels were not affected. However, the results are difficult to compare because of the heterogeneity of the methods of the biomarkers studied. Our review indicates that oxidative stress can be influenced by lifestyle modifications and may be an effective tool for the prevention and management of non-communicable diseases. This review also elucidated the importance of analyzing multiple oxidative stress biomarkers to evaluate oxidative stress, it further highlights the need to conduct long-term lifestyle intervention studies on oxidative stress biomarkers to understand the connection between oxidative stress biomarkers, NCDs and Lifestyle intervention.

## 1 Introduction

Oxidative stress is involved in the pathogenesis of many chronic diseases. (Sharifi-Rad et al., 2020; McGuinness and Higgins, 2021).Oxidative stress (OS) is defined as a state in which the reactive oxygen species (ROS), override cellular antioxidants in the body (Ji and Yeo, 2021). Cells produce ROS as a controlled physiological process, but higher concentration of ROS becomes pathological and leads to oxidative stress and disease. The induction of oxidative stress is an imbalance between the production of radical species and the antioxidant defense systems, which can cause damage to cellular biomolecules, including lipids, proteins, and DNA (Lü et al., 2010; Ghosh and Shcherbik, 2020; Juan et al., 2021). This review examines the role of lifestyle intervention on specific oxidative stress biomarkers; superoxide dismutase, SOD; glutathione, GSH; catalase enzyme, CAT; and malondialdehyde, MDA in chronic diseases.

The antioxidant pathways that form the major line of defense against the OS can be categorically divided into enzymatic and non-enzymatic systems (Sackesen et al., 2008; National Cancer Institute, 2023). Antioxidants are chemicals that prevent the formation of free radicals, interact, and neutralize them, thus preventing them from causing damage, they are also known as “free radical scavengers” (Ziad et al., 2020). Based on their response to general free radical invasion, they can be categorized into first, second, third and even fourth line defense, such as superoxide dismutase (SOD), catalase (CAT), glutathione peroxidase (GPx), ascorbic acid, uric acid, Vitamin E and glutathione (GSH) (Sackesen et al., 2008; Volkova et al., 2012; Mao et al., 2019). Lipid peroxidation LPO is the metabolic process in which reactive oxygen species (ROS) result in the oxidative deterioration of lipids. It is a well-established mechanism of cellular injury (Ayala et al., 2014; Ramana et al., 2014).

SOD and CAT act to suppress or prevent the formation of free radicals or reactive species in cells. They are very fast in neutralizing any molecule with the potential of developing into a free radical or any free radical with the ability to induce the production of other radicals (Zhang et al., 2017; Younus, 2018). GSH is often referred to as scavenging antioxidant. GSH scavenges active radicals to inhibit chain initiation and break chain propagation reactions (Haenen and Bast, 2014). DNA repair enzyme systems (polymerases, glycosylases, and nucleases), proteolytic enzymes (proteinases, proteases, and peptidases) only come into play after free radical damage has occurred. They are *de novo* enzymes which repair the damage caused by free radicals to biomolecules and reconstitute the damaged cell membrane. They are a group of enzymes for repair of damaged DNA, protein, and lipids. They also do a sort of “clean up duty”, they recognize, breakdown and remove oxidized or damaged proteins, DNA and lipids, to prevent their accumulation which can be toxic to body tissues (Ighodaro and Akinloye, 2018). Malondialdehyde (MDA) is a stable end product of lipid peroxidation and therefore can be used as an indirect measure of the cumulative lipid peroxidation (Mao et al., 2019).

If the balance between free radicals and antioxidant levels is disturbed, the resulting oxidative stress is considered a crucial step in the onset and development of pathophysiological changes associated with a variety of inflammatory and non-communicable diseases (NCDs) like; aging, obesity, type 2 diabetes, cardiovascular disease, neurodegenerative diseases, and some types of cancer (Sharifi-Rad et al., 2020; Kelli et al., 2017; Korac et al., 2021; Den et al., 2020; Yilmaz et al., 2020). NCDs are the leading cause of morbidity and mortality worldwide. They are diseases of generally slow progression with clinical symptoms only becoming apparent after considerable cellular damage has occurred in the target tissue, (Seyedsadjadi and Grant, 2020).

In 2021, 71% of deaths, or 41 million, were due to NCD, prevention of NCDs is a global challenge assigned a high priority by the World Health Organization (World Health Organization, 2013; World Health Organization, 2022). NCDs are a result of a combination of various genetic, environmental, and especially lifestyle factors, including smoking, alcohol abuse, unhealthy diets, and physical inactivity (Gbadamosi and Tlou, 2020; Glass, 2021).

Several studies show that timely, well-designed lifestyle interventions, can prevent, improve or delay the progression of NCDs (The Nutrition Source, 2014; Petrides et al., 2019; Gbadamosi and Tlou, 2020; Anand et al., 2022). Numerous intervention studies investigated the effect of single micronutrients or food components on oxidative stress in chronic disease patients. [19, 20]. Many systematic reviews also conclude that a long-term focused intervention is needed to determine whether a lifestyle-based intervention can help reduce oxidative stress (Poljsak, 2011; Liguori et al., 2018; Jiang et al., 2021). A recently published systematic review examined the association of dietary patterns with the biomarkers that characterize oxidative stress. The review included twenty-nine studies, including sixteen observational studies and thirteen intervention studies. Dietary patterns included the Mediterranean diet, the DASH diet, vegetarian and vegan diets, the Daniel Fast diet, the Paleo diet, and a Western diet/fast food diet. The review also found that a plant-based diet resulted in a decrease in several biomarkers characterizing oxidative stress (Aleksandrova et al., 2021).

However, these often show controversial results due to the unstable nature of the oxidative stress biomarkers and the lack of standardized oxidative stress profile analysis (Block et al., 2008; Frijhoff et al., 2015; Ragheb et al., 2020). For this reason, studies with SOD, GSH, CAT, and MDA as biomarkers of OS, focusing on holistic lifestyle interventions (physical, mental, and nutritional) were included in this review.

## 2 Materials and methods

### 2.1 Research question

The research question was: What is the effect of lifestyle interventions on oxidative stress parameters in adults with non-communicable diseases (NCDs)?

### 2.2 Literature searches

The systematic review was guided by the requirements of the Preferred Reporting of Systematic Reviews and Meta-Analyses (PRISMA) statement (Page et al., 2021). The literature search was conducted in the PubMed and Web of Science electronic databases from 08/30/2021 to 11/30/2021. The search was limited to publications from the last 10 years (2012–2021), as the aim was to present the current state of research. Only English-language publications were included. Search terms were limited to titles and abstracts and based on all possible combinations of the following keywords: oxidative stress OR “redox” AND “lifestyle intervention” OR “lifestyle medicine” AND “lifestyle disease” OR “cardiovascular disease” OR “cancer” OR “Alzheimer disease” OR “Parkinson’s disease” OR “diabetes” OR “obesity” OR “metabolic syndrome” OR “non-communicable diseases”.

### 2.3 Eligibility criteria

Studies that examined the effects of lifestyle interventions on changes in oxidative stress biomarkers were included in the systematic review. The inclusion criteria were:

• Articles that measured at least one of the following oxidative stress parameters: GSH, SOD, CAT, and lipid peroxidation (MDA).
• Study participants were required to undergo a lifestyle-based intervention for cardiovascular disease, DMT2, obesity, neurodegenerative disease (Alzheimer’s or Parkinson’s), metabolic syndrome, or cancer.
• Observational studies (cross-sectional, longitudinal, case-control, or cohort studies) and intervention studies (non-randomized and randomized control trials).
• Intervention duration of at least 6 weeks.

The exclusion criteria were:

• Studies that were not conducted in adult participants (<18 years of age).
• No original research (e.g., review articles, editorials).
• Studies who’s full text was not published in English
• Surgical intervention trials.
• Synthetic supplementation.
• Drug studies.
• Animal studies.

### 2.4 Selection of studies

Eligible studies were interventional and observational studies conducted in humans that measured SOD, GSH, CAT, and MDA as markers of oxidative stress. Identified articles were screened for eligibility based on the title and abstract, and duplicates were removed. Two authors (Sarah Husain and Katharina Hillmann) were responsible for retrieving selected articles from four databases and applying inclusion and exclusion criteria to determine eligible studies. If the article was deemed relevant based on the title and abstract, the full text was read and subjected to a second assessment for suitability. Reference lists of included full-text articles and other reviews were reviewed to identify other potentially suitable articles.

### 2.5 Data extraction

A data extraction form was created in Microsoft Word. Regardless of study design, the following information was extracted from the studies: Author, publication year and country, study design, characteristics of participants, details of intervention and control groups, duration of intervention, outcome parameters, and results. Response categories were equipped for open-ended responses. In addition, data on analytical methods and biomarker sample types were collected for each of the selected studies.

### 2.6 Assessment of the risk of bias in the included studies

The risk of bias in the randomized trials was assessed using the Revised Cochrane Risk of Bias Tool for randomized trials (ROB2) (Sterne et al., 2019). Non-randomized studies were assessed separately using the Risk of Bias in Non-randomized Studies - of Intervention (ROBINS-I) assessment tool (Sterne et al., 2016). Two authors (Sarah Husain and Katharina Hillmann) independently assessed the risk of bias. The risk of bias was presented using the ROBONS visualization tool (Moylan and Reid, 2007).

## 3 Results

**FIGURE 1:**
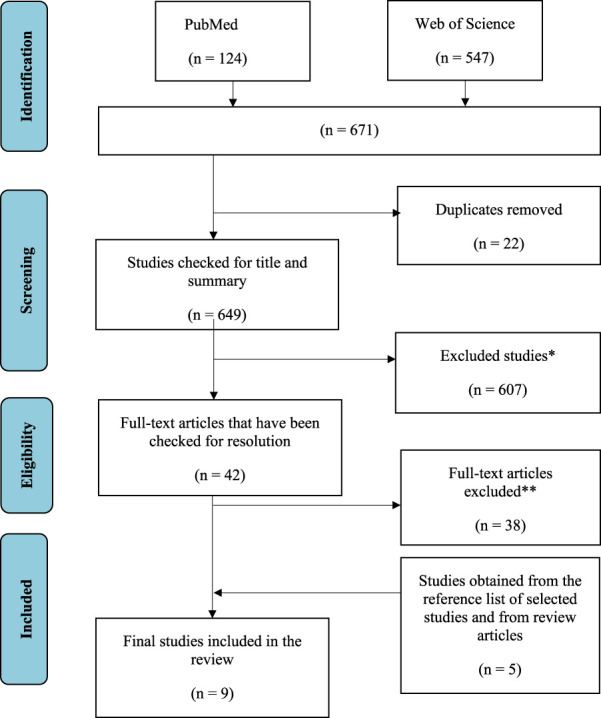
Flowchart of study selection, including identification, review, eligibility, and inclusion of studies (World Health Organization, 2013).

The selection process and the number of articles identified at each step are shown in Figure 1. Six hundred and seventy-one (671) were retrieved in the initial database search. Twenty-two (Yilmaz et al., 2020) duplicates were removed*.* During the initial review of the title and abstract, six hundred and seven (607) titles were excluded. Subsequently, the full texts of forty-two (Luisi et al., 2019) selected articles were reviewed in detail. After the review, an additional thirty-eight (Page et al., 2021) articles were excluded due to eligibility criteria. Therefore, four (Juan et al., 2021) articles were included in this review. In addition, another five (Lü et al., 2010) studies were found by manually searching the reference lists of included studies and matching review articles. Thus, a total of nine articles were included in this systematic review (Figure 1).

*Reasons for exclusion: Irrelevance (n = 165), inappropriate study design (e.g., reviews) (n = 375), inappropriate study population (animals, children) (n = 42), supplements and medications (n = 23), no full text (n = 2).

** Reasons for exclusion: no measurements of oxidative stress biomarkers (n = 28); supplements, medications, and individual foods (n = 8); study protocol only (n = 2).

### 3.1 Characteristics of the included studies

A total of six RCTs (Block et al., 2008; Poljsak, 2011; Liguori et al., 2018; Ragheb et al., 2020; Aleksandrova et al., 2021; Jiang et al., 2021; Anand et al., 2022), one non-randomized intervention study (Luisi et al., 2019), one quasi-experimental intervention study (Ramezanipour et al., 2014), and a prospective longitudinal study (Mirmiran et al., 2018) were included in the systematic review that examined the association between a lifestyle intervention and changes in biomarker concentrations. Study sample sizes varied from 27 to 260 participants for interventional studies (McGeoch et al., 2013; Yadav et al., 2019) and 400 participants for the observational study (Mirmiran et al., 2018). Participants in the intervention studies were between 19 and 80 years old (Ramezanipour et al., 2014; Sureda et al., 2016) and those in the observational study were between 20 and 60 years old (Mirmiran et al., 2018). In one study, only women were included (Ramezanipour et al., 2014). Intervention duration ranged from 8 weeks to 5 years (Sureda et al., 2016; Jaacks et al., 2018). The intervention studies and observational studies were conducted on participants with pre-existing conditions, including metabolic syndrome (Sterne et al., 2019; Ragheb et al., 2020; Aleksandrova et al., 2021; Anand et al., 2022), DMT2 (McGeoch et al., 2013; Dong et al., 2019), and overweight or obesity (Ramezanipour et al., 2014; Jaacks et al., 2018; Luisi et al., 2019). None of the included studies examined changes in oxidative biomarkers in subjects with cardiovascular disease, cancer, or neurodegenerative diseases such as Parkinson’s and Alzheimer’s disease (Table 1; Table 2).

**TABLE 1:** Summary of intervention studies on the relationship between lifestyle intervention and oxidative stress biomarkers.

Author, year	Country	Age (years)	Sample size and study design	Intervention group	Comparison group	Duration	Biomarkers of interests	Main results
Bekkouche et al. (2014) (Bekkouche et al., 2014)	Algeria	56 ± 8^a^	n = 102	84 MetS participants were randomly assigned to the MD, in addition to 30 min of physical activity per day	18 participants Control group (no intervention)	3 months	Plasma: SOD, CAT	The IG has significantly higher SOD and CAT levels as compared to the CG.
			RCT					
			- two-armed					
			- parallel					
Dong et al. (2019) (Dong et al., 2019)	China	51.7 ± 4.9^a^	n = 121	60 Participants received diet and exercise guidelines, and frequent external support	61 Participants received the same treatment as the intervention group but had less frequent external support	6 months	Serum SOD, MDA	The IG group also achieved a greater sustained SOD increase and decrease in MDA as compared to the CG.
			RCT					
			- two-armed					
			- parallel					
Jaacks et al. (2018) (Jaacks et al., 2018)	United States	51.4 ± 6.6	n = 30	Group1: 11 participants received MD	9 participants received a habitual high-fat American-type diet.	8 weeks	Plasma: GSH	Participants in the IG 1; Mediterranean diet arm (n = 11) had improved glutathione (GSH) levels compared to CG (n = 9) at both 4 and 8 weeks
			Non-blinded-RCT	Group 2: 10 participants received a habitual high-fat American-type diet supplemented with fish oil, walnuts, and grape juice				
Luisi et al. (2019) (Luisi et al., 2019)	Italy	52 ± 13.04	n = 36	IG had 18 overweight/obese participants who followed a low-calorie MD (Kcal 1,552 ± 160)	18 normal weight participants followed a typical MD (55%–60% carbohydrates, complex ones, 25%–30% polyunsaturated and monounsaturated fats, 15%–20% proteins)	3 months	Plasma MDA	MDA has significantly decreased within-group comparison after the intervention
			non-randomized intervention study					
			-parallel					
Mc Geoch et al. (2013) (McGeoch et al., 2013)	United Kingdom	60.9 ± 1.23	n = 27	IG 1: Oat-enriched diet standard type 2 diabetic diet.	Baseline (t0) as a comparison group	16 weeks	Plasma MDA	There were no changes in the MDA levels in both the IG as compared to the CG.
			Randomized crossover study	IG 2: reinforced standard dietary advice for type 2 diabetics				
Rameza-nipour et al. (2014) (Ramezanipour et al., 2014)	Iran	19–50	n = 30	30 obese women were recruited in the study and went on a weight loss diet with an energy deficit of 500–1,000 kcal/day through a lower intake of macronutrients and a higher intake of fiber	Baseline (t0) to 3 months within IG	3 months	Plasma SOD, erythrocytes CAT	SOD had no change, but CAT increased after 3 months as compared to baseline
			Quasi-Experimental Study		-no control groups			
Sureda et al. (2016) (Sureda et al., 2016)	Spain	55–80	n = 75	IG 1: 25 participants were giving MeDiet + EVOO	25 participants in the control group received no intervention	5 years	Plasma	Plasma levels of SOD and CAT significantly increased in MeDiet + EVOO and MeDiet + nuts participants as compared to CG.
			RCT					
			- three-armed					
			- parallel					
Yadav et al. (2019) (Yadav et al., 2019)	India	37.7 ± 6.3	n = 260	IG received YBLI, followed by interactive lectures/discussions on yoga, stress management, and nutrition, as well as individual lifestyle counselling	Control participants received the dietary intervention	12 weeks	SOD, MDA	IG group showed a significant decrease in MDA and a significant increase in SOD levels as compared to baseline and a significant increase in SOD and a decrease in MDA levels as compared to CG.
			RCT					
			- two-armed					
			- parallel					

Abbreviations: IG, intervention group; CG, control group; BMI, body mass index; CAT, catalase; GSH, glutathione; MDA, malondialdehyde; SOD, superoxide dismutase; RCT, randomized controlled trial; MD, mediterranean diet; MetS, metabolic syndrome; MeDiet + EVOO: A Mediterranean diet supplemented with extra virgin olive oil; MeDiet + Nuts: A Mediterranean diet supplemented with nuts; YBLI, yoga-based lifestyle intervention.

^a^ Mean age.

^b^ Age in numbers.

**TABLE 2:** Summary of observational studies on the relationship between lifestyle intervention and oxidative stress biomarkers.

Author, year	Country	Age (years)	Sample size and study design	Group one	Group two	Biomarker of interest	Main result
Mirmiran et al. (2018) (Mirmiran et al., 2018)	Iran	20–60	3-year Prospective longitudinal study	1) HDP:High in fruits and dried fruits, olives, low-fat dairy products, poultry, fish, and liquid oils	UDP: Carbonated drinks, fast food, salty snacks, mayonnaise, and organ meats	MDA	1) HDP group showed a significant decrease in MDA levels as compared to the UDP group

Abbreviations: MDA, malondialdehyde; HDP, healthy diet pattern; UDP, unhealthy diet pattern.

### 3.2 Lifestyle interventions

The included studies examined heterogeneous lifestyle interventions. Four interventions were based on dietary interventions, with social support and regular encouragement through knowledge-based advice. Out of these four, three studies examined the Mediterranean diet, with additional consideration of specific foods (Sureda et al., 2016; Jaacks et al., 2018; Luisi et al., 2019). Another dietary intervention was a weight loss diet (Ramezanipour et al., 2014; Mirmiran et al., 2018). The observational study compared a healthy dietary pattern (whole-food plant-based diet) with an unhealthy dietary pattern (more meat and refined food) (Mirmiran et al., 2018). One study supplemented the dietary intervention with 30 min of daily physical activity (Bekkouche et al., 2014) and one with lifestyle recommendations (McGeoch et al., 2013). One study implemented intensive lifestyle modification by providing nutrition and diabetes education, setting dietary and exercise goals, and encouraging participants over a twice-weekly phone call (Dong et al., 2019). Another study also conducted intensive lifestyle modification supplemented with stress management and relaxation. An individualized diet plan was created for each participant, yoga was performed as a physical activity and supplemented with relaxation exercises (Shavasana), education about stress management was provided, and participants also received monthly supervision (Yadav et al., 2019).

In half (n = 4) of the intervention studies, the control groups also received dietary recommendations (Sureda et al., 2016; Dong et al., 2019; Luisi et al., 2019; Yadav et al., 2019). In the other half, the control groups received no intervention and followed their usual diet (McGeoch et al., 2013; Bekkouche et al., 2014; Ramezanipour et al., 2014; Jaacks et al., 2018). In the observational study, the control group followed an unhealthy dietary pattern (Mirmiran et al., 2018). Two intervention studies had healthy, normal-weight individuals as the control group (Bekkouche et al., 2014; Luisi et al., 2019). In contrast, the other studies had the same inclusion criteria for both the intervention group and the control group.

### 3.3 Biomarkers

#### 3.3.1 Oxidative stress

Blood levels of enzymatic and non-enzymatic antioxidants were reported in both the group’s intervention and control, in a total of six articles (Bekkouche et al., 2014; Ramezanipour et al., 2014; Sureda et al., 2016; Jaacks et al., 2018; Dong et al., 2019; Yadav et al., 2019). In one article, GSH activity was measured in reduced form. GSH activity in plasma was measured using high-performance liquid chromatography with fluorescence detection (Jaacks et al., 2018).

Three articles reported on CAT activity. Bekkouche et al. measured CAT activity in plasma, erythrocytes, and platelets (Bekkouche et al., 2014). Ramezanipour et al. examined CAT activity in erythrocytes and Sureda et al. in plasma and total blood (Ramezanipour et al., 2014; Sureda et al., 2016). All three studies used the method designed by Aebi. In this method, CAT activity is measured spectrophotometrically at 240 nm based on the decomposition of hydrogen peroxide in phosphate buffer (Aebi, 2022).

SOD activity was investigated in five articles. The authors used different measurement methods in all five studies. Bekkouche et al. measured SOD activity in plasma, erythrocytes, and platelets by the NADH oxidation method (Fluka/Sigma-Aldrich, Buchs, Switzerland) (Bekkouche et al., 2014). Serum SOD activity was determined by Dong et al. using the SOD assay kit-WST (Dojindo Molecular Technologies, Gaithersburg, MD, United States) (Dong et al., 2019). Ramezanipour et al. measured SOD enzyme activity using the Ransod kit method (cat. No. SD 125, Randox-Ransod, United Kingdom). In this method, SOD activity was determined by measuring the dismutation of superoxide radicals generated by xanthine oxidase and hypoxanthine. The sample type was not specified (Ramezanipour et al., 2014). Sureda et al. determined SOD activity in plasma and total blood by the method of McCord and Fridovich. SOD protein levels were determined by Western blot. Plasma samples were analyzed by SDS-PAGE (Bio-Rad Laboratories, Hercules CA, United States) (Sureda et al., 2016). Yadav et al. determined SOD activity in plasma using a commercially available kit (Cayman Chemical, Ann Arbor, MI, United States) (Yadav et al., 2019).

Lipid peroxidation (LPO) has been investigated in four studies. In the study by Luisi et al., the measurement of LPO in plasma samples was based on the reaction of MDA, the end-product of the process, with 2-thiobarbituric acid (TBA) to form a chromophore that absorbs at 532 nm. Values were expressed in nanomoles of TBA-reactive substances (TBARS) (MDA equivalent) and determined by the Bradford method using an albumin standard curve (Luisi et al., 2019). McGeoch et al. determined MDA from plasma collected in EDTA tubes (Oxi-Select TBARS Assay Kit; Cell Biolabs Inc.) spectrophotometrically after extraction with n-butanol (McGeoch et al., 2013). Mirmiran et al. used the LPO Assay Kit (Abcam, Cambridge, CA, United States) to measure MDA concentration (Mirmiran et al., 2018). Dong et al. did not describe a method for measuring MDA in their publication (Dong et al., 2019).

### 3.4 Effects of lifestyle interventions on oxidative biomarkers

In no study, the oxidative stress biomarkers worsened by lifestyle intervention. Either they improved (increased SOD, GSH, CAT, and decreased MDA) or remained unchanged. It should be noted that the respective studies examined a maximum of two of the included biomarkers (Table 3).

**TABLE 3:** Overview of changes in oxidative biomarkers in subjects depending on lifestyle intervention reported in intervention and observational studies.

Lifestyle intervention	Intervention studies (n = 8)	Observational study (n = 1)
	Biomarker of oxidative stress	Biomarker of oxidative stress
Dietary change	n = 4	n = 1
		MDA: ↓ (Mirmiran et al., 2018)
	SOD: ↑ (Sureda et al., 2016)	
	CAT: ↑ (Ramezanipour et al., 2014; Sureda et al., 2016)	
	MDA: ↓ (Luisi et al., 2019)	
	GSH: ↔ (Jaacks et al., 2018)	
	SOD: ↔ (Ramezanipour et al., 2014)	
Diet change with physical activity	n = 1	-
	SOD: ↑ (Bekkouche et al., 2014)	
	CAT: ↑ (Bekkouche et al., 2014)	
Dietary change with lifestyle recommendations	n = 1	-
	MDA (TBARS): ↔ (McGeoch et al., 2013)	
Intensive lifestyle intervention	n = 1	
	SOD: ↑ (Dong et al., 2019)	
	MDA: ↓ (Dong et al., 2019) -	
Intensive lifestyle intervention with stress management and relaxation	n = 1	-
	SOD: ↔ (Yadav et al., 2019)	

Abbreviations: CAT, catalase; GSH, glutathione; MDA, malondialdehyde; SOD, superoxide dismutase; TBARS, thiobarbituric acid reactive substances.

↓ significant reduction, ↑ a significant increase, ↔ no significant change.

In three out of four intervention studies, dietary change was associated with significant improvement in oxidative stress biomarkers compared to control or baseline levels. Significant increases were found for SOD (*p* < 0.05) and CAT (*p* < 0.05), reflecting lower oxidative stress (Ramezanipour et al., 2014; Sureda et al., 2016). MDA was reduced (*p* < 0.01), implying lower lipid peroxidation (Luisi et al., 2019). Jaacks et al. who examined GSH in the context of lifestyle intervention did not detect a significant increase in the parameter (*p* > 0.05) (Jaacks et al., 2018). Ramezanipour et al. found a positive difference only for CAT, but not for SOD (Ramezanipour et al., 2014).

In the observational study, following a healthy dietary pattern compared to an unhealthy diet was associated with a significant reduction in MDA (Mirmiran et al., 2018).

When the dietary intervention was combined with physical activity, CAT was increased by 27.7% and SOD by 77.7% in platelets compared with baseline (Bekkouche et al., 2014). Nutritional intervention with lifestyle recommendations failed to achieve a significant result in the MDA (LPO) parameter (*p* = 0.264) (McGeoch et al., 2013). In contrast, intensive lifestyle intervention was associated with a significant increase in SOD (*p* < 0.05) and a significant reduction in MDA (*p* < 0.01) (Dong et al., 2019). Contradictory results were shown by Yadav et al., who found no significant increase in the parameter SOD (*p* = 0.238) with an intensive lifestyle intervention supplemented with stress management and relaxation (Yadav et al., 2019).

### 3.5 Relationship between NCDs and oxidative biomarkers

Lifestyle modification reduced oxidative stress in all included NCDs. Each study examined only one of the NCDs.

The majority of included studies examined oxidative stress in subjects with metabolic syndrome (n = 4). Three out of four studies found that lifestyle modification improved antioxidant protection in the form of an increase in SOD and CAT activity, and reduced oxidative stress, expressed by lower levels of MDA (Bekkouche et al., 2014; Sureda et al., 2016; Mirmiran et al., 2018). Only one article failed to find an improvement in antioxidant protection in metabolic syndrome (Yadav et al., 2019).

Three intervention studies examined oxidative stress parameters in overweight and obesity. Ramezanipour et al. found increased activity of CAT but not SOD (Ramezanipour et al., 2014). Luisi et al. reported lower lipid peroxidation in overweight and obese subjects compared to the normal weight control group (Luisi et al., 2019). The GSH parameter remained unchanged (Jaacks et al., 2018).

Two intervention studies investigated oxidative stress biomarkers in DMT2, of which only one study could present significant results. Dong et al. observed both an improvement in SOD activity and a reduction in MDA levels (Dong et al., 2019). In contrast, lifestyle intervention was not associated with lower levels of MDA in the McGeoch et al. study (McGeoch et al., 2013). No studies were found with subjects affected by cardiovascular disease, cancer, and neurodegenerative diseases.

### 3.6 Risk of bias of the included studies

**FIGURE 2:**
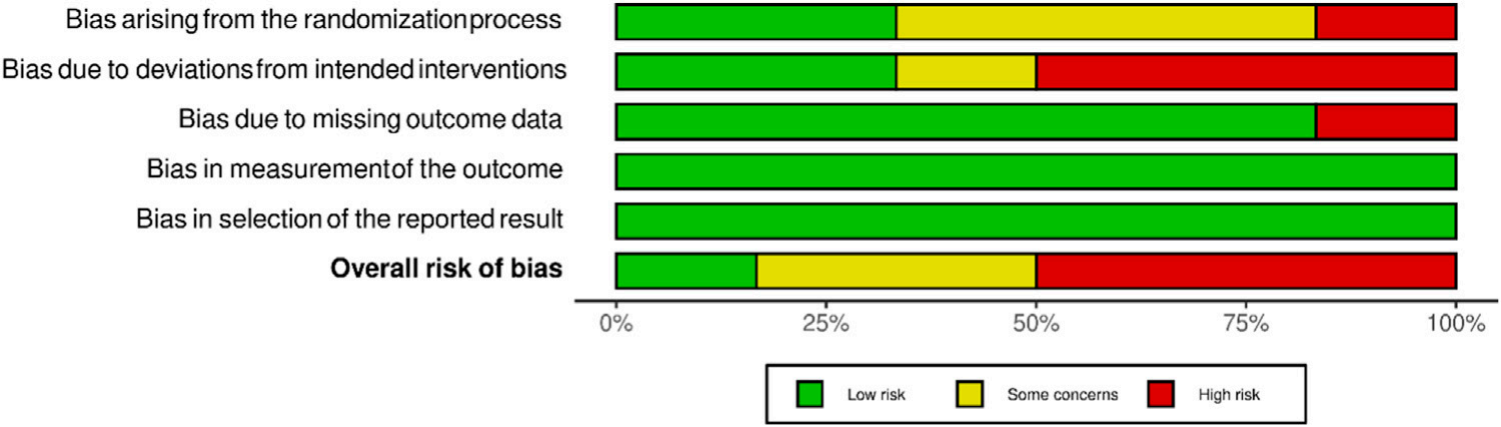
Distribution of assessments of the risk of distortion of RCTs within each bias range (modified according to McGuinnes and Higgins (2020) (Wiley Online Library, 2022b).

Overall, the risk of bias was low in two studies, somewhat concerning in three, and high in four. Five studies did not describe the randomization process. Deviations from the planned intervention also occurred in five studies. One study had a dropout rate of 43% (Bekkouche et al., 2014) and was classified as having a high risk of bias due to missing outcome data. The domains “bias in the measurement of the outcome” and “bias in the selection of the reported outcome” had 100% negligible risk among the studies assessed (Figure 2).

## 4 Discussion

This systematic review shows the positive effects of lifestyle interventions on biomarkers of oxidative stress in NCDs. Six of nine studies (Bekkouche et al., 2014; Ramezanipour et al., 2014; Sureda et al., 2016; Jaacks et al., 2018; Mirmiran et al., 2018; Dong et al., 2019; Luisi et al., 2019) reported increased activity of antioxidants SOD and CAT or decreased lipid peroxidation measured as MDA. A recently published systematic review that summarized the results from 29 studies among which 16 were observational and 13 were interventional studies concluded that a plant-based diet improved several biomarkers of oxidative stress (Aleksandrova et al., 2021).

In addition, improvement in oxidative stress was found in all NCDs studied (metabolic syndrome, overweight and obesity, and DMT2). According to current research, this work is the first systematic review summarizing the effects of different lifestyle interventions on biomarkers of oxidative stress in the context of NCDs.

### 4.1 Lifestyle interventions

The included studies examined lifestyle interventions using natural foods without synthetic supplements. Dietary changes in the studies were often based on plant-based foods (though with high consumption of fruits, vegetables, legumes, whole grains, nuts, and olive oil) (Bekkouche et al., 2014; Sureda et al., 2016; Luisi et al., 2019). High consumption of fruits and vegetables and whole grains, with increased intake of dietary fiber, is associated with a lower risk of cardiovascular disease, breast cancer, DMT2, cancer, and cardiovascular disease mortality (Partula et al., 2020). The components in fruits and vegetables appear to reduce oxidative stress. Diet rich in fruits and vegetables appears to reduce oxidative stress as they are rich in bioactive phytochemicals. Phytochemicals can be literally referred to as ‘plant-chemicals.’ They are the non-nutritive chemical components of plants that possess numerous health benefits and disease prevention properties (Freese et al., 2002; Dragsted et al., 2004; Nwozo et al., 2023). Oral supplementation with non-enzymatic antioxidants such as GSH has not been successful due to the poor bioavailability of GSH (Allen and Bradley, 2011). No robust data was found for enzymatic antioxidant supplementation and its effect on systemic oxidative stress or lifestyle diseases. The results from the studies indicate that dietary changes through foods high in antioxidants, such as fruits and vegetables can improve our oxidative stress status with or without supplementation. The health benefits of fruits, vegetables, whole grains, and other plant foods cannot be achieved or mimicked by dietary supplements in the form of tables or pills without fully upstanding the balanced natural combination and profiles of phytochemicals present in fruits, vegetables, and whole grain (Liu, 2013; Samtiya et al., 2021).

Another explanation for the reduction of oxidative stress by lifestyle intervention is that weight loss reduces oxidative stress (Himbert et al., 2017). Some of the different lifestyle interventions resulted in weight reduction in study participants (Bekkouche et al., 2014; Ramezanipour et al., 2014; Dong et al., 2019; Luisi et al., 2019). Except for Yadav et al. (Yadav et al., 2019), these studies resulted in an improvement in oxidative stress. These results are consistent with previous studies (Rector et al., 2007; Moreto et al., 2015).

### 4.2 Oxidative stress parameters

SOD levels are not affected by lifestyle interventions in NCDs patients. The statistical non-significance of the improvement in SOD levels between the intervention group and the control group can be explained by the fact that at the beginning of the study, the two groups had a strong difference in SOD levels (*p* = 0.071). In the 36–45 age group, the YBLI showed a significantly greater increase in SOD levels after 12 weeks compared to the control group (*p*-value not shown). This was not shown in the 20–35-year-old group. When the age groups were considered together, no significant results were shown (Yadav et al., 2019). This suggests that age plays a vital role in oxidative stress. The participants were too young to show the effects of oxidative stress and therefore no improvement in antioxidant enzymes associated with lifestyle change could be detected. One reason why SOD remained unchanged in the study by Ramezanipour et al. may be that a 10% weight reduction in obese individuals was not sufficient to increase SOD levels (Ramezanipour et al., 2014). Another reason could be the methodology used to study SOD levels. Since there is no gold standard, different methods and sample types have been used to measure SOD activity.

Interestingly, the studies uniformly showed a significant increase in CAT levels in subjects who performed lifestyle intervention (Bekkouche et al., 2014; Ramezanipour et al., 2014; Sureda et al., 2016). Since all studies used the same measurement method according to Aebi et al. (Aebi, 2022), CAT values are comparable from the different studies apart from the different sampling procedures.

The only study that measured GSH levels reported no changes in GSH after lifestyle intervention (Jaacks et al., 2018). GSH is an important intracellular non-protein thiol whose concentration is strongly affected by hemodialysis of plasma and serum samples as well as by storage time (Giustarini et al., 2004; Aquilano et al., 2014; Giustarini and Colombo, 2022).

This review demonstrated negative associations between lifestyle intervention and lipid peroxidation in both intervention and observational studies. A plausible mechanism for this could be that the dietary changes and healthy eating patterns implemented in the studies were high in omega-3 fatty acids. Studies show that omega-3 fatty acids decrease lipid peroxidation (Akrami et al., 2018; Heshmati et al., 2019). In addition, changes in MDA levels appear to be influenced by changes in glucose levels. This may be explained by the fact that hyperglycemia-induced stress leads to the production of advanced glycation end products (AGEs). AGEs are formed by glycation and are associated with an increase in oxidative stress since they mediate the production of reactive oxygen species (ROS). The interaction of AGEs with the receptor for AGEs (RAGE) enhances oxidative stress through ROS production by NADPH oxidases inside the mitochondria (Schmidt et al., 1994; Goldin et al., 2006; Cepas et al., 2020). Therefore, control of glucose metabolism is effective in reducing glycation processes. Due to the formation of AGEs, hyperglycemia, and insulin resistance predispose to the occurrence of oxidative stress. Exercise training activates molecular mechanisms that improve insulin sensitivity and glucose tolerance in peripheral tissues. Consequently, this reduces cellular glucotoxicity and exposure to lipid peroxidation (Moreto et al., 2015).

In addition to the differences in intervention, analytical methods, and biological sample types described above, there are other reasons why studies demonstrated different changes or no improvement in oxidative stress after lifestyle intervention. First, the duration of a few weeks might not be sufficient to reach statistical significance. A systematic review also concludes that a longer-term intervention is needed to determine whether a diet-based intervention can help reduce oxidative stress (Nwozo et al., 2023). Secondly, the number of participants in each study may not have been large enough to achieve statistically significant power. Three of the four studies that found no or only partial changes in oxidative stress parameters had small sample sizes of 27–30 participants (McGeoch et al., 2013; Ramezanipour et al., 2014; Jaacks et al., 2018). In addition, the control groups of the intervention studies are heterogeneous. On one hand, participants in the control group did not receive any intervention in four intervention studies (Poljsak, 2011; Liguori et al., 2018; Page et al., 2021; Anand et al., 2022) and on the other hand, the control group in four other intervention studies performed a similar intervention (Sureda et al., 2016; Dong et al., 2019; Luisi et al., 2019; Yadav et al., 2019). Additionally, the inclusion criteria were either same for all the participants or for the participants of the control group who did not have a disease. For example, in the study by Bekkouche et al.; participants in the control group showed significantly higher levels of oxidative stress markers SOD and CAT (*p* < 0.005) and the intervention group showed a significantly lower levels of clinical markers (*p* < 0.005) as compared to baseline (Bekkouche et al., 2014).

None of the studies measured the overall oxidative stress profile or the combination of these four parameters (SOD, CAT, GSH, MDA). Four of the nine studies examined a maximum of two of the included oxidative stress biomarkers (Aleksandrova et al., 2021; Jiang et al., 2021; Page et al., 2021; Anand et al., 2022). The remaining five studies analyzed only one relevant oxidative stress biomarker (McGeoch et al., 2013; Jaacks et al., 2018; Mirmiran et al., 2018; Luisi et al., 2019; Yadav et al., 2019). Due to this, it is not possible to draw reliable conclusions about the effect of lifestyle intervention on biomarkers of oxidative stress in subjects with NCDs. It is important to determine not only the effect of a lifestyle change on a specific antioxidant but to examine a profile of oxidative stress biomarkers, otherwise, the oxidative status of an individual cannot be assessed accurately (Kochlik et al., 2017).

No other systematic review or meta-analyses examining the effects of lifestyle changes on oxidative stress were found. However, two similar systematic reviews were identified. Both studies also excluded dietary supplements, tablets, and powders and were based on whole-food components. However, these specialized in the effects of diet or dietary patterns rather than overall lifestyle changes. Furthermore, these did not focus on specific oxidation-related biomarkers and included both healthy and diseased participants (Vetrani et al., 2013; Aleksandrova et al., 2021).

A recently published systematic review examined the association of dietary patterns with the biomarkers of oxidative stress. This systematic review included twenty-nine studies, including sixteen observational studies and thirteen intervention studies. Dietary patterns included the Mediterranean diet, the DASH diet, vegetarian and vegan diets, the Daniel Fast diet, the Paleo diet, and a Western diet/fast food. The review found that a plant-based diet improved several biomarkers of oxidative stress (Aleksandrova et al., 2021).

One further systematic review examined whether the diet and its components can alter oxidative stress *in vivo*. For this purpose, nine observational studies and forty-nine intervention studies were analyzed. The review found that the consumption of low caloric diet rich in fruits and vegetables positively affect oxidative biomarkers, as the preventive effects of plant products have been largely allocated to their high content of various phytochemicals such as flavonoids, betalains and carotenoids and the overall high antioxidant capacity (Freese et al., 2002; Di Gioia et al., 2020). In contrast, saturated fatty acids and alcoholic beverages worsen oxidative status (Vetrani et al., 2013).

### 4.3 Strengths and limitations of the systematic review

Strengths of this systematic review include the use of a standardized method for conducting a systematic literature search (PRISMA), which was conducted independently by two researchers. The study focused on a variety of lifestyle interventions including the holistic nature of lifestyle change. In contrast, the selection of biomarkers was defined in advance to increase the comparability of the studies and to be able to present an overall profile of oxidative stress. Only diseases of major importance in terms of lifestyle were included. The review includes published studies from the last 10 years, allowing latest trends in research to be presented.

Limitations of the systematic review should also be considered. The specificity of the biomarkers is not only beneficial but also has the disadvantage that many studies had to be excluded that investigated other biomarkers for oxidative-stress might have provided important insights. Assessment of individual oxidative stress biomarkers may not be representative of the overall magnitude of oxidative stress. However, no study assessed a combination of multiple oxidative stress biomarkers as endpoints in their analyses, so the overall profile could not be assessed in the systematic review. Overall, the quality of the intervention studies is also low to moderate, allowing for some potential biases. In addition, no assessment tool for bias was applied to the observational study, as no specific assessment tool is available for observational studies according to the Cochrane Collaboration (Sterne et al., 2016; Sterne et al., 2019). Most studies had short study durations and small study populations, so the power of the studies is low.

### 4.4 Implications for future research

Many studies that explored the effects of lifestyle changes on lifestyle-based diseases have already been published. Future studies should also include the oxidative stress components because current research suggests that oxidative stress plays a significant role in the pathogenesis of lifestyle-based diseases. Furthermore, a gold standard for oxidative stress analysis should be established e.g., a complete oxidative stress profile or analysis of multiple biomarkers. The study duration of most of the included studies is relatively short. Therefore, further studies with a longer intervention and follow-up period should be conducted to explore the long-term effects of lifestyle intervention. Also, larger studies with more participants should be conducted to obtain clearer results.

## 5 Conclusion

This systematic review provides evidence that lifestyle modifications can improve oxidative stress biomarkers and can be an effective tool for the prevention and treatment of non-communicable diseases. However, the systematic review shows that there is a lack of long-term lifestyle-based intervention studies investigating an association between lifestyle and oxidative stress in non-communicable diseases with multiple biomarkers of oxidative stress. Therefore, further studies are needed to confirm and extend these findings.
